# The impact of face masks on the recall of spoken sentencesa)

**DOI:** 10.1121/10.0002951

**Published:** 2021-01-06

**Authors:** Thanh Lan Truong, Sara D. Beck, Andrea Weber

**Affiliations:** English Department, Eberhard Karls University, Tübingen, Germany

## Abstract

The effect of face covering masks on listeners' recall of spoken sentences was investigated. Thirty-two German native listeners watched video recordings of a native speaker producing German sentences with and without a face mask, and then completed a cued-recall task. Listeners recalled significantly fewer words when the sentences had been spoken with a face mask. This might suggest that face masks increase processing demands, which in turn leaves fewer resources for encoding speech in memory. The result is also informative for policy-makers during the COVID-19 pandemic, regarding the impact of face masks on oral communication.

## I. INTRODUCTION

Understanding spoken language requires the translation from speech signal to meaning: phonetic, lexical, and syntactic information must be extracted, and linguistic meaning in sentences must be composed. As adult listeners, we typically carry out these complex mental tasks with astonishing ease and speed. However, processing becomes cognitively more demanding when the speech signal is acoustically degraded or ambiguous (e.g., Refs. 1, 2). Increased listening effort in adverse conditions has also been shown to affect higher-level cognitive processing downstream, such as memory encoding. That is, listeners are worse at recognizing which words they have heard before and at recalling exact lexical items when the speech input is degraded, for example, in casual or accented speech or in noisy environments (e.g., Refs. 3–5).

In this study, we examined the effect of wearing a face mask on subsequent recall of spoken sentences. A speaker's lip and jaw movements contain linguistic information. For example, lip closure correlates with a bilabial place of articulation for the stop consonants /p/ and /b/, and the openness of the jaw is correlated with the height of vowels (more open jaw for the vowel /a/ and less open jaw for /i/). This visual information is complementary to the auditory signal, and information from both domains is automatically integrated during speech perception (e.g., Ref. 6). Concealing visual speech information with a mask could therefore result in a decrease in encoding performance. At the same time, mask material could degrade the acoustic signal by dampening it and acting as a low-pass filter. While some studies indeed found effects of various types of mouth and face coverings on speech acoustics (e.g., Refs. 7, 8), others found the effects to be negligible (e.g., Ref. 9). We tested the effect of face masks on memory for spoken language using a cross-modal cued-recall task (see Ref. 5). German native listeners watched video recordings of a native speaker producing sentences (e.g., *Die Köchin hilft montags armen Kindern*, “the cook helps on Mondays poor children”) with and without a face mask. After a block of sentences, participants were cued in orthographic form with the sentence beginnings (e.g., *Die Köchin hilft montags*, “the cook helps on Mondays”) and had to fill in the missing final two words (e.g., *armen Kindern*, “poor children”). Similar to other forms of signal degradation, we expected recall rates to be lower for sentences produced with a face mask.

Face masks in public places have been mandatory in many countries during the COVID-19 global pandemic and have become part of our daily lives. There is currently a need to better understand the possible impact of wearing a mask, not only on physical and psychological comfort, but also on verbal communication. Testing the retention of spoken information is one aspect of this.

## II. METHODS

### A. Participants

Thirty-two native German listeners between the ages of 20 and 37 years participated in the study (mean: 23.8; 28 females). All participants indicated normal hearing and vision. They were recruited via social media and university email, and electronically signed written informed consent and filled out a brief language background questionnaire. For monetary compensation, participants were given the opportunity to participate in a lottery.

### B. Stimuli

The stimuli consisted of 48 German sentences, modelled after the *Oldenburger Satztest*.^10^ All sentences began with a determiner and a noun, followed by a verb, an adverb, an adjective, and a noun. The sentences were not highly predictable in order to reduce the facilitatory influence of context, and to ensure a more thorough processing of the input.^11^ All words were of high lexical frequency, and each content word occurred only once in the stimuli.

**FIG. 1.**
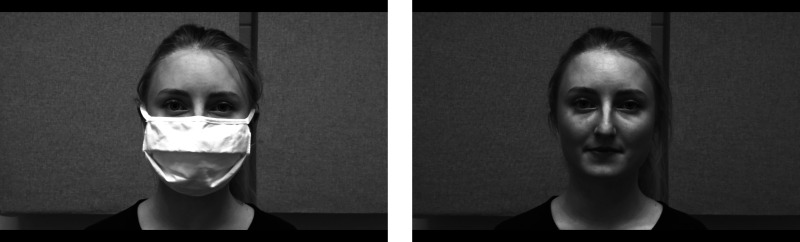
Representative screen shots for video recordings with and without a face mask. Videos were presented in color in the experiment.

A 22-year-old female native speaker of German was video recorded producing all sentences with and without a face mask (see Fig. 1). Recordings were made in a sound-attenuated room with a high-quality, stationary RØDE microphone at a sampling rate of 44 kHz and a Sony DSC-Hx90 camera recorder with video resolution parameters set to Full HD 1920 × 1080, which was positioned to capture the speaker's head and shoulders. The face mask was made of two layers of fabric: The inner layer consisted of a thin fleece layer, and the outer layer was cotton. The speaker was instructed to produce all sentences at a normal speaking rate without hesitations or pauses and to not speak more clearly or loudly when wearing the mask. Unmodified, natural sentence recordings without a mask were on average 3172 ms long and with a mask 3253 ms (*t* = −1.39).^12^ Spectral analysis (RMS power) revealed no difference between sentences with (56.6 db) and without a face mask (56.7 db) (*t* = −0.28).

### C. Procedure

The experiment was implemented with the online software *SurveyGizmoLLC* (surveygizmo.com, 2020). Participants were asked to wear headphones and participated online. The experiment started with two practice sentences and continued with the 48 experimental sentences, divided into eight blocks of six sentences. Sentence order was randomized once, and half of the participants watched the videos in the reverse order. The presence of a face mask was blocked, and blocks alternated between the mask and no-mask condition. The order of mask condition was counterbalanced, and sentences were presented with an ISI of 2500 ms. The self-paced cued-recall task followed each block. For this task, sentences were presented up to the adverb orthographically on the screen (e.g., *Die Köchin hilft montags*, “the cook helps on Mondays”), and participants were asked to type in the missing two final words (e.g., *armen Kindern*, “poor children”) on their keyboard. For each participant, there was a total of 96 keywords (2 keywords in each of the 48 sentences) to be recalled. All sentence beginnings of a block were available at once, in the order of block presentation, and participants could choose in which order they typed their responses.

### D. Results

**FIG. 2.**
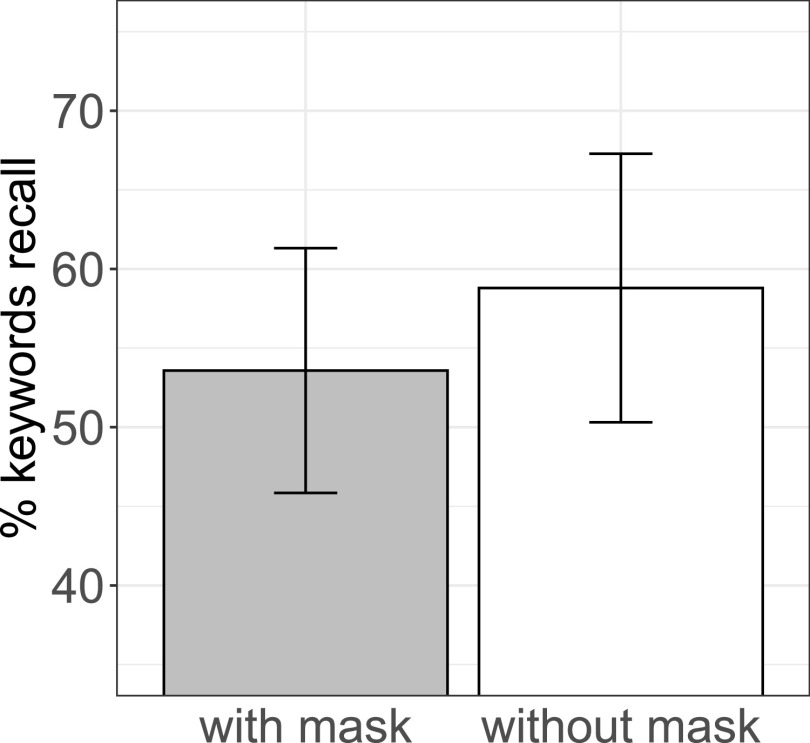
Average percentage of keywords recalled correctly for sentence recordings with and without a face mask. The vertical bars represent standard errors.

Each recalled keyword was scored by the first author and a research assistant as either correct (1) or incorrect (2) (see Fig. 2). Approximately 70% of all responses that were categorized as incorrect, had been omissions. In the remaining 30% of incorrected responses, a variety of error types was observed: the majority were responses that were unrelated in form and meaning to the keywords (e.g., “schwarze Schuhe,” *black shoes*, for “staubige Kissen,” *dusty pillows*); a much smaller number of responses were closely semantically related (e.g., “Ringe,” *rings*, for “Kreise,” *circles*); only few responses were phonetic errors involving a single sound change, i.e., a substitution, insertion, or deletion (e.g., “Schweine,” *pigs*, for “Steine,” *stones*) or typos (e.g., the nonword “Lmpen” for “Lampen,” *lamps*). To assess the effect of face masks on listeners' keyword recall, a logistic mixed-effects regression model^13^ was implemented using the lme4 package^14^ in r (version 4.0.2).^15^ Accuracy was modeled as binary categorical (success vs failure). Face mask (mask vs no mask) and block (8 blocks) were entered as fixed effects. To test linear and quadratic effects of block, orthogonal polynomials was used.^16^ Items and participants were included as random crossed effects,^17^ with random intercepts and random slopes for both. The analysis showed a difference in keyword recall when the speaker was not wearing a mask compared to when she was wearing a mask (*b =* −0.29, *SE* = 0.12, *p* = 0.017). There was no significant interaction.

## III. CONCLUSION

In a cued-recall experiment, native adult listeners recalled fewer words when the speaker had been wearing a face mask than when she had not been wearing one. This result suggests that processing speech produced with a face mask leaves fewer cognitive resources available for storing spoken information in memory. Face masks both conceal visual speech information and can degrade the acoustic signal (e.g., Refs. 7, 8). While the present study was not set out to tease apart the reasons for why face masks decrease encoding performance, we have some indications that neither the acoustic signal nor speech perception were affected much by the mask. A lack of a difference in RMS values between the mask and no-mask condition indicates that, at least spectrally, the two conditions did not differ. In a small *post hoc* experiment, we also asked an additional 12 participants to write down the keywords after individual sentences, rather than after blocks of sentences, rendering the task into an assessment of intelligibility. Performance was overall highly correct and did not differ between the mask (98.95% correct) and no mask condition (99.3% correct). Thus at least for a clear speech style, recorded in a quiet environment, and a cotton mask, the missing visual cues rather than decreased intelligibility seem to have been the main factor causing a decrease in encoding performance. Future experiments investigating the intelligibility of speech with masks in noise can, however, help to clarify this point.

In order to get a fuller understanding of the impact of face masks on memory for spoken language, different participant groups and speakers must be tested next. For example, non-native listeners and children can be expected to have more difficulties in perceiving spoken language than native adults have due to their incomplete mastery of the target language. For these listener groups, removing visual cues with a mask might have an even stronger impeding effect on memory (e.g., Ref. 18). Also, speakers with varying language experience (e.g., non-natives and children) can deviate noticeably in their pronunciation from the target norms of a language. In such cases, native adult listeners typically rely even more on visual speech cues (e.g., Ref. 19), and concealing these cues with a face mask can be expected to intensify the negative effect on the encoding of spoken information.

For native adult listeners and native speech, the present results already indicate that face masks can impede memory for what has been said. This finding should have implications for communication in various situations, for example, in classrooms and doctor's offices where remembering spoken information is crucial.

## References

[1] M. Ernestus , H. Baayen , and R. Schreuder , “ The recognition of reduced word forms,” Brain Lang. 81, 162–173 (2002). 10.1006/brln.2001.2514 12081389

[2] M. J. Witteman , A. Weber , and J. M. McQueen , “ Tolerance for inconsistency in foreign-accented speech,” Psychon. Bull. Rev. 21, 512–519 (2014). 10.3758/s13423-013-0519-8 24234167

[3] R. C. Gilbert , B. Chandrasekaran , and R. Smiljanić , “ Recognition memory in noise for speech of varying intelligibility,” J. Acoust. Soc. Am. 135(1), 389–399 (2014). 10.1121/1.4838975 24437779

[4] A.-K. Grohe and A. Weber , “ Memory advantage for produced words and familiar native accents,” J. Cogn. Psychol. 30, 570–587 (2018). 10.1080/20445911.2018.1499659

[5] S. Keerstock and R. Smiljanić , “ Clear speech improves listeners' recall,” J. Acoust. Soc. Am. 146(6), 4604–4610 (2019). 10.1121/1.5141372 31893679

[6] A. Jesse and D. W. Massaro , “ The temporal distribution of information in audiovisual spoken-word identification,” Atten. Percept. Psychophys. 72(1), 209–225 (2010). 10.3758/APP.72.1.209 20045890

[7] R. M. Corey , U. Jones , and A. C. Singer , “ Acoustic effects of medical, cloth, and transparent face masks on speech signals,” J. Acoust. Soc. Am. 148(4), 2371–2375 (2020). 10.1121/10.0002279 33138498 PMC7857499

[8] L. L. Mendel , J. A. Gardino , and S. R. Atcherson , “ Speech understanding using surgical masks: A problem in health care?,” J. Am. Acad. Audio. 19(9), 686–695 (2008). 10.3766/jaaa.19.9.4 19418708

[9] C. Llamas , P. Harrison , D. Donnely , and D. Watt , “ Effects of different types of face coverings on speech acoustics and intelligibility,” York Pap. Ling. 9, 80–104 (2009).

[10] Oldenburger Satztest , *Handbuch und Hintergrundwissen* (*Manual and Background Knowledge*) (HörTech GmbH, Oldenburg, Germany, 2000).

[11] J. Rommers and K. D. Federmeier , “ Predictability's aftermath: Downstream consequences of word predictability as revealed by repetition effects,” Cortex 101, 16–30 (2018). 10.1016/j.cortex.2017.12.018 29414458 PMC5869124

[12] See supplementary material at https://www.scitation.org/doi/suppl/10.1121/10.0002951 for video example of a sentence recorded with and without a face mask.

[13] T. F. Jaeger , “ Categorical data analysis: Away from ANOVAs (transformation or not) and towards logit mixed models,” J. Mem. Lang. 59, 434–446 (2008). 10.1016/j.jml.2007.11.007 19884961 PMC2613284

[14] D. Bates , M. Mächler , B. Bolker , and S. Walker , “ Fitting linear mixed-effects models using lme4,” J. Stat. Softw. 67(1), 1–48 (2015). 10.18637/jss.v067.i01

[15] R Core Team, “ R: A language environment for statistical computing. R Foundation for Statistical Computing,” https://www.r-project.org/ (Last viewed June 2020).

[16] D. Mirman , *Growth Curve Analysis and Visualization Using R* ( Taylor Francis, London, 2017).

[17] R. H. Baayen , D. Davidson , and D. Bates , “ Mixed effects modeling with crossed random effects for subjects and items,” J. Mem. Lang. 59, 390–412 (2008). 10.1016/j.jml.2007.12.005

[18] S. Keerstock and R. Smiljanić , “ Effects of intelligibility on within-and cross-modal sentence recognition memory for native and non-native listeners,” J. Acoust. Soc. Am. 144(5), 2871–2881 (2018). 10.1121/1.5078589 30522310

[19] Z. Xi , H.-G. Yi , and B. Chandrasekaran , “ Nonnative audiovisual speech perception in noise: Dissociable effects of the speaker and listener,” PLoS One 9(12), e114439 (2014). 10.1371/journal.pone.0114439 25474650 PMC4256416

